# Identification of a Tumor Microenvironment-Related Eight-Gene Signature for Predicting Prognosis in Lower-Grade Gliomas

**DOI:** 10.3389/fgene.2019.01143

**Published:** 2019-11-15

**Authors:** Jun Su, Wenyong Long, Qianquan Ma, Kai Xiao, Yang Li, Qun Xiao, Gang Peng, Jian Yuan, Qing Liu

**Affiliations:** ^1^Department of Neurosurgery in Xiangya Hospital, Central South University, Changsha, China; ^2^Department of Neurosurgery in Peking University Third Hospital, Peking University, Beijing, China; ^3^Institute of Skull Base Surgery & Neuro-oncology at Hunan, Changsha, China

**Keywords:** lower-grade gliomas, tumor microenvironment, weighted gene co-expression network analysis, prognosis, immune cells infiltration

## Abstract

Lower-grade gliomas (LrGG), characterized by invasiveness and heterogeneity, remain incurable with current therapies. Clinicopathological features provide insufficient information to guide optimal individual treatment and cannot predict prognosis completely. Recently, an increasing amount of studies indicate that the tumor microenvironment plays a pivotal role in tumor malignancy and treatment responses. However, studies focusing on the tumor microenvironment (TME) of LrGG are still limited. In this study, taking advantage of the currently popular computational methods for estimating the infiltration of tumor-associated normal cells in tumor samples and using weighted gene co-expression network analysis, we screened the co-expressed gene modules associated with the TME and further identified the prognostic hub genes in these modules. Furthermore, eight prognostic hub genes (*ARHGDIB, CLIC1*, *OAS3*, *PDIA4*, *PARP9*, *STAT1*, *TAP2*, and *TAGLN2*) were selected to construct a prognostic risk score model using the least absolute shrinkage and selection operator method. Univariate and multivariate Cox regression analysis demonstrated that the risk score was an independent prognostic factor for LrGG. Moreover, time-dependent ROC curves indicated that our model had favorable efficiency in predicting both short- and long-term prognosis in LrGG patients, and the stratified survival analysis demonstrated that our model had prognostic value for different subgroups of LrGG patients. Additionally, our model had potential value for predicting the sensitivity of LrGG patients to radio- and chemotherapy. Besides, differential expression analysis showed that the eight genes were aberrantly expressed in LrGG compared to normal brain tissue. Correlation analysis revealed that the expression of the eight genes was significantly associated with the infiltration levels of six types of immune cells in LrGG. In summary, the TME-related eight-gene signature was significantly associated with the prognosis of LrGG patients. They might act as potential prognostic biomarkers for LrGG patients, offer better stratification for future clinical trials, and be candidate targets for immunotherapy, thus deserving further investigation.

## Introduction

Lower-grade gliomas (LrGG) are infiltrative and heterogeneous brain neoplasms that include World Health Organization (WHO) grade II and III diffuse gliomas (Patel et al., 2017). Because of their highly invasive characteristics, complete neurosurgical resection is unachievable for most patients. The residual tumor results in recurrence and malignant progression at variable intervals; some of these tumors recur and even progress to glioblastoma (WHO grade IV) within months, whereas others remain indolent for years (van den Bent et al., 2011; Zhou et al., 2017). The current treatment modalities for LrGG typically involve neurosurgical resection, observation, chemotherapy, and/or radiotherapy. However, none of these treatment options are curative for this disease (Kumthekar et al., 2015). The main purpose of treatment is to delay tumor progression and improve quality of life (Duffau and Taillandier, 2015). However, due to considerable heterogeneity between LrGG, an optimal treatment strategy against this disease at the individual level still remains a challenge (Duffau, 2018). From this perspective, it is necessary to develop reliable approaches for identifying subsets of patients at high risk of deterioration and to find novel molecular targets for the development of effective therapeutic strategies.

Recently, the tumor microenvironment (TME), including tumor-associated normal epithelial and stromal cells, immune cells, and vascular cells, has been observed to participate in cancer biology and has received increasing amounts of attention. Increasing studies show that a highly heterogeneous TME plays a substantial role in tumor malignancy and treatment responses and critically impacts immunotherapeutic strategies (Platten et al., 2014; Li et al., 2017; Ma et al., 2018). For example, epithelial and stromal cells participate in tumor growth, malignant progression, and treatment resistance (Kalluri and Zeisberg, 2006; Straussman et al., 2012). Infiltrating immune cells, including macrophages, dendritic cells, mast cells, natural killer cells, and lymphocytes, also exhibit tumor-promoting features in a context-dependent manner (Fridman et al., 2012; Yoshihara et al., 2013). Thus, strengthening the knowledge of TME and highlighting the mechanism underlying its effects will ultimately contribute to the diagnosis and treatment of gliomas. Considering the importance of TME in cancer treatment, many computational methods have been developed to estimate the infiltration of tumor-associated normal cells in tumor samples using genomic approaches such as ESTIMATE (Estimation of STromal and Immune cells in MAlignant Tumours using Expression data), TIMER (Tumor Immune Estimation Resource), and CIBERSORT (Cell-type Identification By Estimating Relative Subsets Of RNA Transcripts) (Yoshihara et al., 2013; Newman et al., 2015; Li T. et al., 2017). These methods can facilitate a comprehensive understanding of tumor biology and the development of robust predictive models in long-term tumor management. In fact, increasing studies have successfully applied these algorithms to various cancers including prostate cancer, breast cancer, and colon cancer. (Ali et al., 2016; Alonso et al., 2017). However, application of these algorithms to explore the cellular networks underlying the glioma TME are still limited, especially in LrGG.

In the present study, through construction of gene co-expression networks using a weight gene co-expression network analysis (WGCNA) and the identification of TME-related gene co-expression modules, we aimed to identify the prognostic genes involved in the TME of LrGG and to set up an effective prognostic model for LrGG. Our findings might provide novel insights into the TME of LrGG and provide important clues for predicting the prognosis of LrGG and for the selection of individualized therapies for LrGG patients.

## Materials and Methods

### Data Sets

RNA sequencing data of The Cancer Genome Atlas (TCGA) LrGG (530 samples, 516 patients) containing RSEM normalized data and log2(RSEM+1) transformed data were obtained from the Broad GDAC firehose (http://gdac.broadinstitute.org/) and the University of California, Santa Cruz, Xena browser (UCSC Xena, https://xenabrowser.net/), respectively. The clinical data of LrGG (516 patients, five of which had no survival data) were also downloaded from UCSC Xena. The stromal score (positively correlating with the presence of stroma in tumor tissue) and immune score (positively correlating with the level of immune cells infiltrations in tumor tissue) of TCGA LrGG dataset generated using the ESTIMATE algorithm were downloaded from https://bioinformatics.mdanderson.org/estimate/. Another cohort of LrGG patients (181 patients, nine of which had no survival data) was obtained from the Chinese Glioma Genome Atlas (CGGA, www.cgga.org.cn/), and their mRNA sequencing data (FPKM) and clinical data were downloaded. In the present study, the TCGA cohort was used as training data and the CGGA cohort was used for validation. The information of LrGG patients including survival information from both TCGA and CGGA datasets is shown in **Table 1**.

**Table 1:** Clinical characteristics of LrGG with survival information in TCGA and CGGA datasets.

	TCGA	CGGA
No. of patients	511	172
age (mean, year)	40.03	40.42
Gender		
Male	283	105
Female	228	67
Censor		
alive	386	122
death	125	50
WHO grade		
G II	246	105
G III	264	67
unknown	1	–
IDH status		
WT	90	44
Mut	411	128
unknown	10	–
MGMT promoter methylation		–
methylated	416	–
unmethylated	85	–
unknown	10	–
karnofsky performance score (KPS)		–
>70	254	–
≤70	47	–
unknown	210	–

### Weighted Gene Co-Expression Network Analysis

Weighted gene co-expression network analysis (WGCNA) was performed using the “WGCNA” package (Langfelder and Horvath, 2008) in R language, version 3.5.3 (www.r-project.org). According to the instructions for WGCNA, the RSEM data were used for subsequent analysis. Prior to network construction, we checked the RNA sequencing data and removed the genes and samples with too many missing values as well as obvious outlier samples. Then, based on the criterion of approximate scale-free topology, we chose the soft thresholding power β to construct unsigned co-expression networks. Co-expression similarity was calculated, defined as S_ij_, which equals the absolute Pearson’s correlation coefficient between gene i and j. Then, the similarity matrix was transformed to the weighted adjacency matrix, defined as Aij = S_ij_^β^. The topological overlap matrix (TOM) was derived from the weighted adjacency matrix. Based on the TOM, a hierarchical clustering tree (dendrogram) of genes was produced using a hierarchical clustering method. Finally, we used the standard method, Dynamic Tree Cut, to identify co-expression gene modules with a deep split level of two and a minimum module size of 30. A height cut of 0.25, corresponding to a correlation of 0.75, was used to merge similar modules. We calculated the Gene Significance (GS) and Module Membership (MM) to evaluate the gene relationship between ESTIMATE scores (immune score and stromal score) and each module. Modules significantly associated with the two scores (p value < 0.05) were screened out, and the genes in these modules were selected for further analysis.

### Functional Enrichment Analysis

Functional enrichment analyses, including gene ontology (GO) analysis comprising cellular component (CC), molecular function (MF), and biological process (BP), and Kyoto Encyclopedia of Genes and Genomes (KEGG) pathway analysis, were performed using the clusterProfiler package in R language (Yu et al., 2012). The reference gene set for analyses is whole transcriptome as RefSeq transcript set. The P value adjusted by Benjamini and Hochberg method.

### Hub Gene Identification and Module Visualization

Hub genes were identified using the R package WGCNA. The thresholds for identifying hub genes in each target module were |MM| > 0.8 and |GS| > 0.2. Finally, we obtained 54, 15, and 8 hub genes in the green, salmon, and magenta module, respectively. Visualization of the targeted modules was performed using Cytoscape (Shannon et al., 2003), an open source software for visualizing molecular interaction networks.

### Protein-Protein Interaction Analysis

Protein-protein interaction (PPI) analysis of survival-related genes was performed using an online software, The Search Tool for the Retrieval of Interacting Genes (STRING, https://string-db.org/) (Szklarczyk et al., 2019), and the PPI network was visualized using Cytoscape.

### Survival Analysis

Kaplan–Meier survival analysis and the Cox proportional hazard model were performed using R language packages (survival, survminer, and ggplot2).

### Construction of Prognostic Risk Score Model *via* the Least Absolute Shrinkage and Selection Operator Method

Least absolute shrinkage and selection operator (LASSO) is a penalization method to shrink and select variates for regression (Tibshirani, 1997). It has been widely used in the genetic data analyses of various cancers, including TCGA data (Zhao et al., 2015). In this article, univariate Cox regression analysis was first performed to identify 74 prognostic hub genes of three TME-related gene co-expression modules in TCGA cohort. As one (*LILRB4*) of these 74 genes was missing in the CGGA LrGG cohort, the remaining 73 genes were selected for LASSO Cox regression analysis in TCGA LrGG cohort, and finally, 8 prognostic genes were selected to the construct a prognostic risk model. The formula for calculating the risk score was as follows: risk score = (-0.1880 * expression level of A*RHGDIB*) + (0.3131 * expression level of *CLIC1*) + (0.0546 * expression level of *OAS3*) + (0.2527 * expression level of *PARP9*) + (0.1052 * expression level of *STAT1*) + (0.1776 * expression level of *PDIA4*) + (0.2479 * expression level of *TAGLN2*) + (-0.2525 * expression level of *TAP2*).

### Time Dependent Receiver Operating Characteristic Curve Analysis

The Receiver Operating Characteristic (ROC) curve analysis is a widely used method for assessing the sensitivity and specificity of a continuous diagnostic marker for a binary disease variable (Kamarudin et al., 2017). Time-dependent ROC curve analysis was used to evaluate the performance, including the predictive accuracy and sensitivity, of our prognostic model within 1 year, 3 years, and 5 years of OS using the R package, survivalROC (Heagerty et al., 2000). The optimal cutoff of the risk score was calculated using the Youden index. In the TCGA cohort, the optimal cutoffs of the risk score for 1 year, 3 years, and 5 years of OS were 7.61036429, 7.37154483, and 7.37154483, respectively. In the CGGA cohort, the optimal cutoffs for 1 year, 3 years, and 5 years of OS were 1.790661239, 1.790661239, and 1.736271505, respectively. In both TCGA and CGGA cohorts, the patients were divided into high- and low-risk groups based on the optimal cutoff for 5 years of OS, respectively. In TCGA LrGG cohort, 121 patients were classified in the high-risk group and 390 patients belonged to the low-risk group. In the CCGA LrGG cohort, there were 64 patients in the high-risk group and 108 patients in the low-risk group.

### Differential Expression Analysis

Differential expression analysis of eight genes within our model was performed using the online database Gene Expression Profiling Interactive Analysis (GEPIA). GEPIA (Tang et al., 2017) is an interactive web platform for gene expression analysis, which integrates the cancer samples from the TCGA database and normal samples from the GTEx database.

### Analysis of Immune Infiltration

TIMER is a comprehensive resource for the analysis of immune infiltrates across various cancers (https://cistrome.shinyapps.io/timer/) (Li T. et al., 2017). An estimation of immune cells, including B cells, CD4+ T cells, CD8+ T cells, neutrophils, macrophages, and dendritic cells, in TCGA LrGG was performed using TIMER. We analyzed the correlation between the risk model system (risk score and eight genes within the model) and infiltrating levels of six immune cells using an R package (psych). Spearman’s correlation was calculated by the corr.test function in psych package, and scatter plots were generated using the ggplot2 package in the R language. We used the following standard to describe the strength of correlation for the absolute value of r: absolute values between 0 and 0.3 indicate a weak correlation; absolute values between 0.3 and 0.7 indicate a moderate correlation; absolute values between 0.7 and 1.0 indicate a strong correlation (Akoglu, 2018).

## Results

### Immune Scores and Stromal Scores Are Significantly Associated With LrGG Subtypes and Prognosis

**Figure 1:**
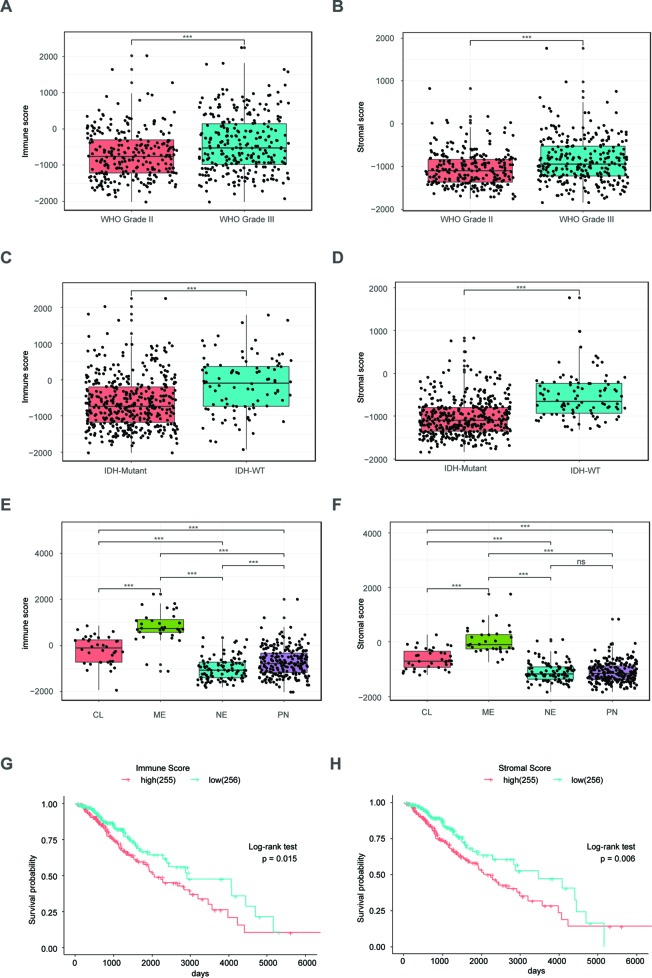
Immune score and stromal score correlate with LrGG subtype and outcome. **(A**–**B)** Both immune score and stromal score positively associated with the WHO grade in LrGG. **(C**–**D)** Both Immune score and stromal score associated with the IDH status in LrGG. **(E**–**F)** Both immune score and stromal score corelated with the transcriptome subtypes of LrGG. **(G**–**H)** Both immune score and stromal score associated with the prognosis of LrGG. *** P 0.001, ns P 0.05, **(A**–**F)** t-test, **(G**–**H)** Log-rank test.

The ESTIMATE algorithm is based on a single sample Gene Set Enrichment Analysis, and its predictive ability has been well validated (Yoshihara et al., 2013). The stromal score and immune score positively reflect the presence of stroma cells and immune cells, respectively, in tumor tissues. First, we plotted the distribution of immune scores and stromal scores in the TCGA LrGG cohort. The results showed that both immune scores and stromal scores in grade III glioma were significantly higher than those in grade II glioma (**Figures 1A, B**). To reveal the molecular expression patterns of immune and stromal scores, we compared both immune scores and stromal scores in IDH (isocitrate dehydrogenase) subtypes [including IDH wildtype (IDH-Wt) and IDH Mutant (IDH-Mut)] as well as four transcriptome subtypes (including mesenchymal, classical, neural, and preneural) of LrGG. The results showed that both immune scores and stromal scores in IDH-Mut subtype samples were significantly lower than those in IDH-Wt subtype samples (**Figures 1C, D**). The immune scores of four transcriptome subtypes were significantly different and those of mesenchymal subtype patients were the highest, followed by the classical subtype, neural subtype, and proneural subtype (**Figure 1E**). Separately, the stromal scores of transcriptome subtypes were similar to the results of immune scores, except there was no significant difference between the stromal scores of neural and proneural subtype samples (**Figure 1F**). To determine the potentially clinical value of the immune score and the stromal score for patients with LrGG, the Kaplan–Meier survival analysis was performed. Based on the median of immune score and stromal scores, LrGG were divided into two groups, the high group (score > median) and low group (score ≤ median). The result showed that patients in the low immune score group had a significantly longer overall survival (OS) compared to patients in the high immune score group (**Figure 1G**). Similarly, cases with a low stromal score had better prognosis than those with a high stromal score (**Figure 1H**). In summary, these results indicate that both immune scores and stromal scores correlate significantly with LrGG subtypes, and that high scores predict a relatively poor prognosis for LrGG patients.

### Construction of Weight Co-Expression Network Using WGCNA

**Figure 2:**
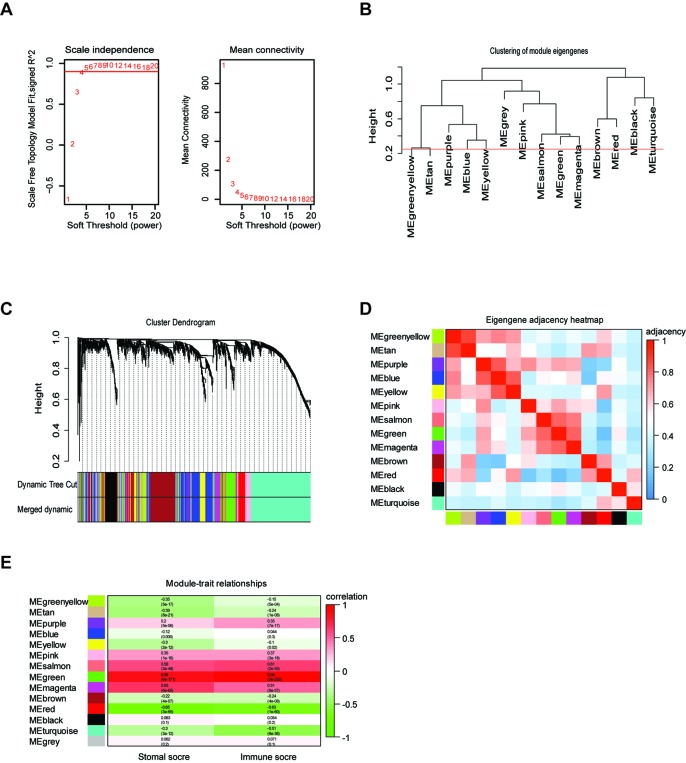
Weighted gene co-expression network of LrGG. **(A)** Analysis of network topology for various soft-thresholding powers and identification of suit soft -thresholding power to construct a scale-free network. **(B)** Clustering dendrogram of consensus module eigengenes. The red line represents merging threshold and modules with a correlation coefficient more than 0.75 were merged. **(C)** Hierarchical cluster analysis dendrogram used to detect co-expression models along with corresponding color assignments in LrGG. **(D)** The eigengene adjacency heatmap of 13 co-expression models. The eigengene adjacency A_IJ_ = (1 + cor (E_I_, E_J_))/2. The table is color-coded by adjacency according to the color legend, which decreased in size from red to blue. **(E)** Correlation between the gene modules and immune scores as well as stromal score. Rows correspond to module eigengenes, columns correspond to traits. Each cell contains the corresponding correlation and p-value. The table is color-coded by correlation according to the color legend, which decreased in size from red to green.

To provide system-level insights and high sensitivity to small fold changes in genes, WGCNA can be applied to high-throughput microarray or RNA-seq datasets and can describe the correlation between gene modules and external conditions (Pei et al., 2017). Prior to WGCNA, the RNA sequencing data of TCGA LrGG (530 samples) was constructed into a matrix with gene IDs as row names and sample barcodes as column names. Further, genes and samples with too many missing values were removed and the genes were then ranked by variance from large to small; the top 5,000 genes were selected for WGCNA. After one outlier was identified and removed (**Supplementary Figure S1**), we constructed the co-expression network based on the remaining 529 samples with 5,000 genes *via* the WGCNA package. As shown in **Figure 2A**, β = 4, which is the lowest power for which the scale-free topology fit index reached 0.90, was selected to calculate the adjacencies. Based on TOM, a hierarchical clustering tree (dendrogram) of genes was produced using the hierarchical clustering function. After merging similar modules, we obtained 13 gene modules (**Figures 2B–D**) with size ranging from 37 to 1,426 genes. We assigned each co-expression module an arbitrary color for reference: black, blue, brown, green, greenyellow, magenta, pink, purple, red, salmon, tan, turquoise, and yellowturquoise. These modules contained 262, 791, 647, 358, 115, 206, 220, 133, 348, 37, 70, 1,426, and 371 genes, respectively. As a single group, the non-co-expressed group was designated as ‘gray’ based on the WGCNA developer’s rationale, and 16 genes in this group were removed.

### Identifying the TME-Associated Modules and Prognostic hub Genes of LrGG

**Figure 3:**
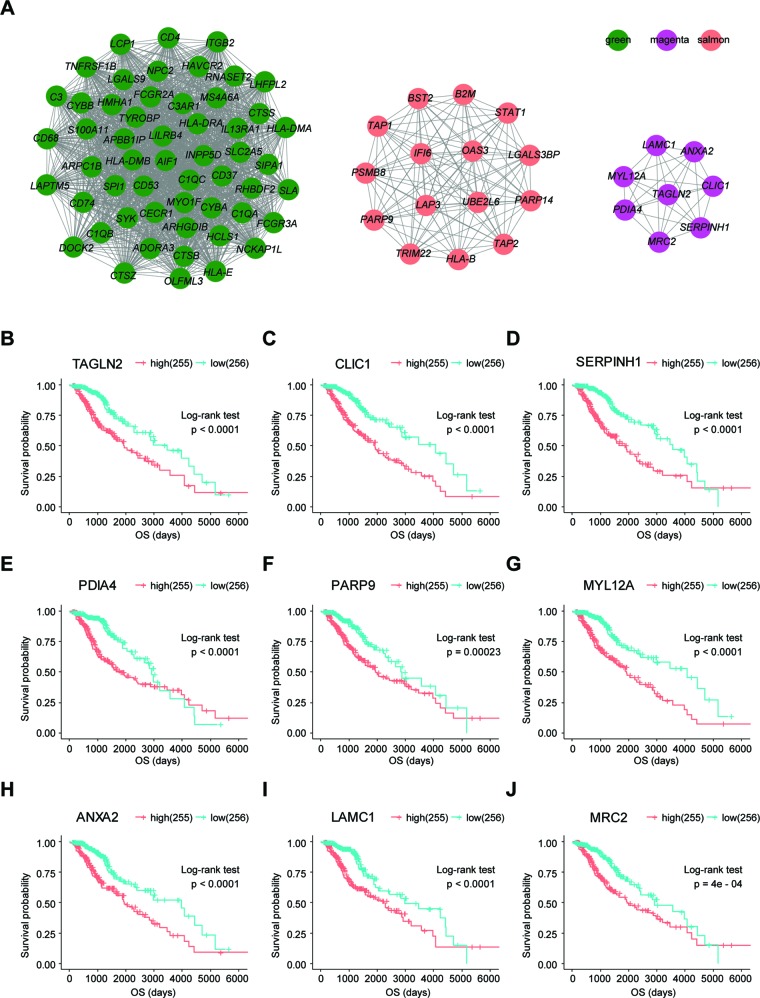
Visualization of hub genes in three modules and Kaplan-Meier survival curves of the top nine prognostic genes. **(A)** Visualization of hub genes in the green, magenta, and salmon modules, respectively, based on weight. **(B**–**J)** Kaplan-Meier survival curves for the top nine prognostic genes ranked by p value from small to large (grouped by median value of the gene expression level).

To determine whether any module was associated with immune scores and stromal scores, a principal components analysis was performed to generate the module eigengene (ME). MEs provide single-column summary measures of the overall gene expression level of each co-expression module. Then we calculated the correlations between the scores and each co-expression module. The results showed that 11 modules including greenyellow, tan, purple, yellow, pink, salmon, green, magenta, brown, red, and turquoise were correlated with immune scores and stromal scores (**Figure 2E**, p value < 0.05). Furthermore, GO enrichment analysis was performed to identify TME-related modules from the 11 abovementioned modules. The results revealed that the three co-expression modules, green, salmon, and magenta, were significantly associated with the TME (**Supplementary Figure S2**, **Supplementary Table S1**). Consistently, these three modules belong to the same branch of the clustering dendrogram of module eigengenes (**Figure 2B**). However, the remaining modules were rarely correlated with TME (**Supplementary Table S2**). In a co-expression network, hub genes are defined as genes inside co-expression modules that have high connectivity (Langfelder and Horvath, 2008). Hub genes are highly co-expressed with other genes and play key roles in critical pathways (Uddin and Singh, 2017). To identify the hub genes of the three target modules, intramodular connectivity and module membership for all genes in each module were calculated using the WGCNA package. Based on the preset threshold (|MM| > 0.8 and |GS| > 0.2), we identified 54, 15, and 8 hub genes in the green, salmon, and magenta module, respectively. The co-expression network of the hub genes in each module was visualized using Cytoscape based on the weight, which indicated that these hub genes were highly connected (**Figure 3A**). To identify the prognostic genes of LrGG patients from the 77 hub genes, univariate Cox regression analysis was performed and the result showed that 74 genes were significantly correlated with the OS of LrGG patients in TCGA cohort (p value < 0.05, **Table 2**), and the Kaplan-Meier survival curves of the top nine significant genes are shown in **Figures 3B–J**. In addition, 70 of the 74 genes were well validated in the LrGG cohort from the CGGA, an independent glioma database (**Table 2**).

**Table 2:** Univariate Cox analysis of 77 hub genes.

Hub genes	TCGA	P value (Wald test)	CGGA	P value (Wald test)
	HR (95% CI)		HR (95% CI)	
TAGLN2	1.88 (1.65-2.15)	4.03E-21	2.54 (1.97-3.27)	4.29E-13
CLIC1	2.05 (1.76-2.4)	2.19E-19	1.98 (1.62-2.41)	2.76E-11
PDIA4	2.5 (1.98-3.15)	1.37E-14	2.89 (2.15-3.9)	2.56E-12
SERPINH1	1.78 (1.53-2.06)	2.18E-14	3.29 (2.3-4.71)	6.56E-11
PARP9	2.05 (1.7-2.46)	3.09E-14	3.22 (1.91-5.44)	1.19E-05
MYL12A	2.17 (1.77-2.66)	1.33E-13	2.3 (1.74-3.04)	4.05E-09
ANXA2	1.56 (1.39-1.76)	2.42E-13	6.11 (3.57-10.4)	3.83E-11
LAMC1	1.87 (1.58-2.22)	2.94E-13	1.99 (1.61-2.46)	2.63E-10
MRC2	1.73 (1.49-2.02)	1.47E-12	1.5 (1.31-1.73)	9.24E-09
STAT1	1.85 (1.56-2.2)	4.17E-12	2.21 (1.65-2.98)	1.43E-07
S100A11	1.65 (1.43-1.91)	8.17E-12	1.91 (1.56-2.34)	3.86E-10
OAS3	1.56 (1.37-1.78)	1.69E-11	1.57 (1.23-2)	3.17E-04
LGALS3BP	2.02 (1.64-2.49)	4.26E-11	2.99 (2.16-4.12)	2.81E-11
PARP14	1.85 (1.54-2.22)	6.28E-11	1.99 (1.48-2.66)	4.32E-06
IFI6	1.45 (1.29-1.62)	1.24E-10	1.59 (1.28-1.97)	2.31E-05
CECR1	1.78 (1.49-2.14)	2.40E-10	65.5 (3.92-1090)	3.61E-03
LAP3	2.15 (1.68-2.74)	7.16E-10	3.36 (2.35-4.81)	3.19E-11
BST2	1.6 (1.38-1.87)	1.36E-09	1.85 (1.49-2.3)	2.00E-08
TRIM22	1.69 (1.42-2)	1.48E-09	3.78 (2-7.15)	4.46E-05
TAP1	1.77 (1.44-2.17)	6.05E-08	1.94 (1.46-2.56)	3.64E-06
LHFPL2	1.65 (1.37-2)	2.26E-07	2.2 (1.56-3.11)	7.35E-06
HLA.B	1.56 (1.31-1.84)	2.65E-07	2.03 (1.58-2.62)	4.57E-08
HLA.DRA	1.32 (1.19-1.47)	3.14E-07	1.67 (1.38-2.01)	1.07E-07
FCGR2A	1.43 (1.24-1.64)	4.44E-07	4.11 (2.09-8.08)	4.16E-05
CD74	1.36 (1.2-1.53)	5.62E-07	1.66 (1.35-2.03)	1.10E-06
OLFML3	1.53 (1.29-1.81)	1.37E-06	5.65 (3.13-10.2)	9.36E-09
IL13RA1	1.64 (1.34-2)	1.41E-06	1.78 (1.25-2.55)	0.00142
B2M	1.7 (1.37-2.12)	2.18E-06	2.41 (1.7-3.42)	7.45E-07
PSMB8	1.7 (1.36-2.11)	2.27E-06	2.7 (1.83-3.99)	5.12E-07
HLA.DMB	1.39 (1.21-1.59)	2.71E-06	1.86 (1.46-2.36)	5.49E-07
HCLS1	1.41 (1.22-1.64)	4.25E-06	1.86 (1.4-2.46)	1.43E-05
CTSZ	1.57 (1.29-1.91)	6.24E-06	2.05 (1.56-2.68)	2.34E-07
HLA.DMA	1.37 (1.2-1.58)	6.41E-06	1.88 (1.49-2.37)	1.17E-07
HLA.E	1.71 (1.35-2.15)	7.07E-06	1.85 (1.27-2.69)	0.00134
FCGR3A	1.27 (1.14-1.4)	7.41E-06	81300 (191–34700000)	2.53E-04
MYO1F	1.44 (1.23-1.69)	7.49E-06	2.14 (1.53-3.01)	1.09E-05
CTSB	1.98 (1.46-2.7)	1.37E-05	3.06 (1.87-4.99)	7.58E-06
SLA	1.42 (1.21-1.67)	1.77E-05	2.88 (1.26-6.57)	0.0119
INPP5D	1.49 (1.24-1.8)	2.19E-05	48.8 (2.49-958)	0.0105
CTSS	1.38 (1.18-1.62)	5.25E-05	3.02 (1.81-5.05)	2.49E-05
SIPA1	1.51 (1.23-1.86)	8.46E-05	85.2 (9.07-800)	1.00E-04
MS4A6A	1.26 (1.12-1.41)	9.43E-05	11.5 (4.11-32.1)	3.26E-06
NPC2	1.56 (1.25-1.96)	0.000107	2.35 (1.7-3.24)	2.10E-07
ARPC1B	1.44 (1.2-1.74)	0.000109	2.22 (1.64-3.02)	3.29E-07
LGALS9	1.38 (1.17-1.63)	0.000151	2.48 (1.42-4.31)	0.00133
C1QB	1.3 (1.13-1.49)	0.000152	1.85 (1.47-2.33)	2.16E-07
RNASET2	1.4 (1.17-1.67)	0.000185	1.76 (1.28-2.43)	0.000529
HAVCR2	1.33 (1.14-1.56)	0.000257	1.68 (1.26-2.25)	0.000486
CD37	1.32 (1.13-1.52)	0.000289	1.8 (1.25-2.59)	0.00146
CYBA	1.35 (1.15-1.59)	0.000296	1.87 (1.43-2.44)	4.36E-06
ITGB2	1.3 (1.12-1.5)	0.000366	3.15 (1.92-5.18)	5.67E-06
LCP1	1.35 (1.14-1.6)	0.000445	1.68 (1.23-2.29)	0.00112
C3	1.24 (1.1-1.39)	0.000501	1.62 (1.33-1.97)	1.35E-06
TAP2	1.67 (1.25-2.22)	0.00055	2.21 (1.32-3.7)	0.00251
ARHGDIB	1.41 (1.16-1.71)	0.000563	2.08 (1.55-2.78)	8.82E-07
C1QC	1.28 (1.11-1.48)	0.000726	1.76 (1.36-2.29)	1.90E-05
CD53	1.31 (1.12-1.54)	0.000852	11100 (4.27-28800000)	0.0202
C1QA	1.26 (1.1-1.45)	0.000954	1.89 (1.49-2.4)	1.80E-07
SPI1	1.3 (1.11-1.53)	0.00101	2.39 (1.64-3.48)	5.46E-06
TNFRSF1B	1.35 (1.13-1.61)	0.00109	1.76 (1.25-2.5)	0.00132
CD68	1.31 (1.11-1.54)	0.0011	2.03 (1.49-2.78)	8.69E-06
TYROBP	1.28 (1.1-1.5)	0.00179	2.94 (1.74-4.96)	5.30E-05
LAPTM5	1.29 (1.1-1.51)	0.00187	1.77 (1.36-2.31)	2.12E-05
RHBDF2	1.28 (1.09-1.49)	0.00204	4.18 (0.874-20)	0.0733
HMHA1	1.37 (1.1-1.71)	0.00578	5.4 (0.66-44.1)	0.116
NCKAP1L	1.23 (1.06-1.43)	0.006	8.49 (3.04-23.7)	4.56E-05
ADORA3	1.24 (1.06-1.44)	0.00611	1.37 (1.02-1.83)	0.0375
CD4	1.27 (1.06-1.52)	0.00883	2.37 (1.47-3.84)	0.000441
APBB1IP	1.2 (1.04-1.38)	0.0116	1.42 (1.06-1.9)	0.0178
LILRB4	1.18 (1.03-1.36)	0.0208	Null	Null
SYK	1.19 (1.02-1.39)	0.0232	8.09e+13 (4.32e-15-1.51e+42)	0.335
C3AR1	1.18 (1.02-1.35)	0.0237	1.38 (1.05-1.81)	0.0228
CYBB	1.16 (1.02-1.33)	0.0263	1.59 (1.21-2.1)	0.000967
DOCK2	1.17 (1.01-1.35)	0.0352	1.72 (1.18-2.49)	0.00445
UBE2L6	1.44 (0.974-2.12)	0.0673	–	–
SLC2A5	1.1 (0.963-1.27)	0.154	–	–
AIF1	1.1 (0.943-1.28)	0.228	–	–

### Functional Enrichment and PPI Analysis of Prognostic Genes

**Figure 4:**
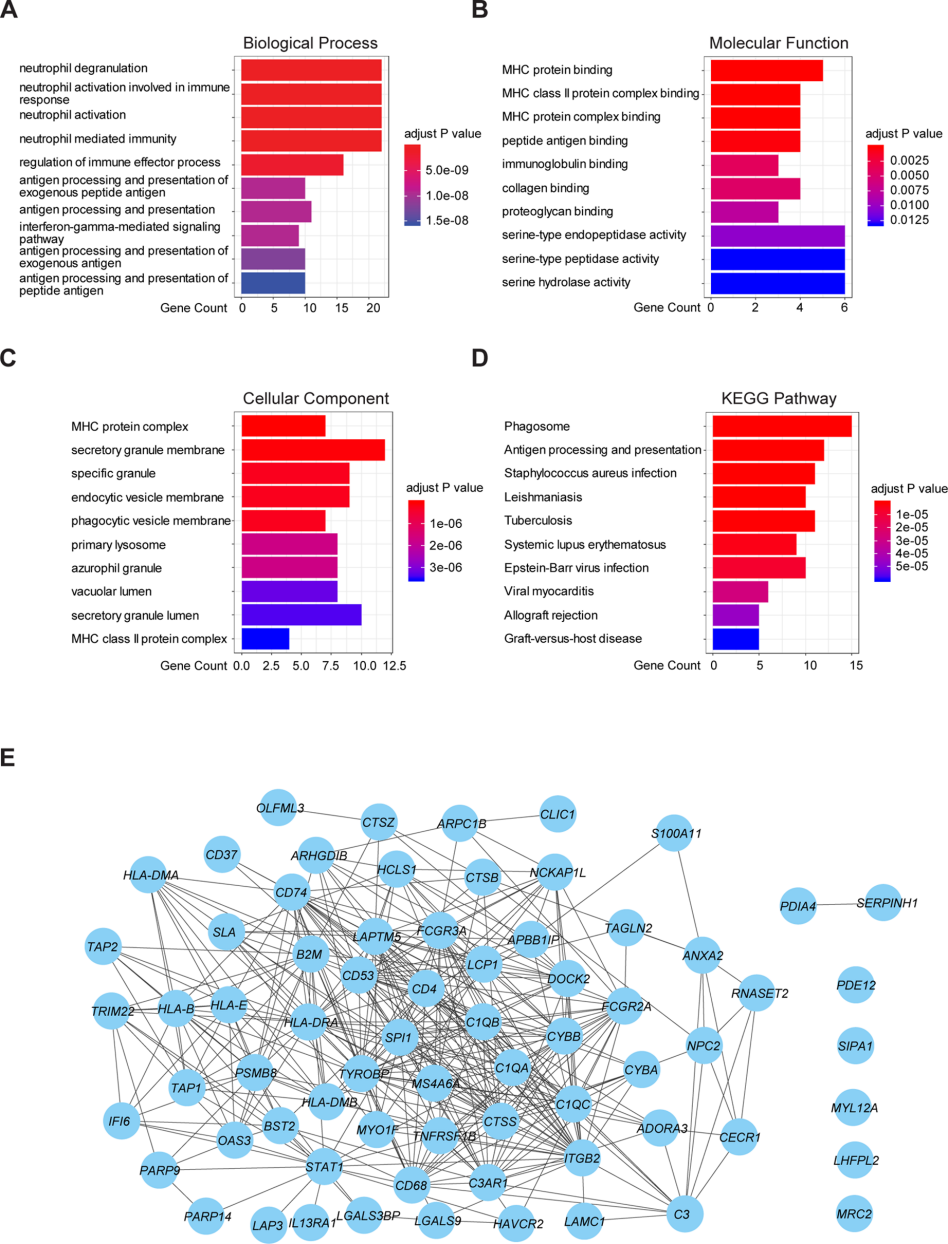
Functional enrichment analysis and PPI network of 70 prognostic genes in TCGA cohort. **(A)** The top 10 biological process terms of GO enrichment analysis of 70 prognostic genes. **(B)** The top 10 molecular function terms of GO enrichment analysis of 70 prognostic genes. **(C)** The top 10 cellular component terms of GO enrichment analysis of 70 prognostic genes. **(D)** KEGG pathway analysis for 70 prognostic genes and visualization of the top 10 terms. **(E)** PPI network of the 70 prognostic genes was visualized by Cytoscape.

To further understand the underlying mechanism of these prognostic genes, functional enrichment analysis was performed. GO enrichment analysis showed the 70 prognostic genes significantly associated with immune-related terms. For GO analysis, a total of 366 terms of biological process (BP), 81 terms of cellular component (CC), and 21 terms of molecular function (MF) were identified to be statistically significant (adjusted p value < 0.05, Benjamini and Hochberg method, **Supplementary Table S3**). The top terms of BP included neutrophil degranulation, activation, neutrophil mediated immunity, and antigen processing and presentation (**Figure 4A**). MF indicated enrichments predominantly involved in MHC protein binding, cell adhesion molecule binding, and antigen binding (**Figure 4B**). As for CC, these genes showed significant enrichment in the MHC protein complex, vacuolar lumen, and collagen-containing extracellular matrix (**Figure 4C**). Additionally, KEGG pathway analysis revealed that these genes were significantly enriched in immune related pathways such as antigen processing and presentation, Th1 and Th2 cell differentiation, Th17 cell differentiation, and Leukocyte transendothelial migration (**Figure 4D**, **Supplementary Table S3**). To further understand the interaction among these genes, PPI networks (which contained 70 nodes and 366 edges) were constructed based on the STRING database (**Figure 4E**). This network contained 70 nodes and 366 edges and was highly connected.

### Construction and Validation of Prognostic Risk Score Model for LrGG

**Figure 5:**
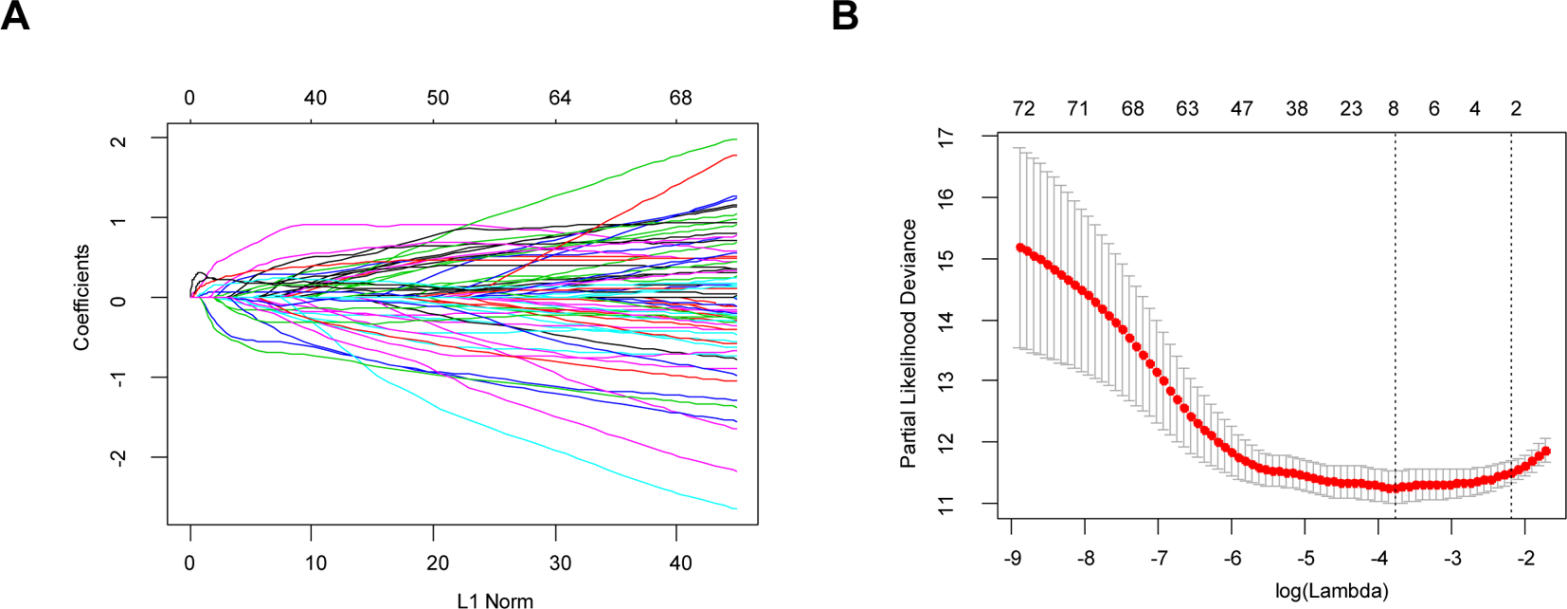
Identification of independent prognostic TME-related genes by LASSO regression. **(A)** LASSO coefficient profiles of 73 prognostic genes. **(B)** Ten-time cross-validation for tuning parameter selection in the LASSO model.

**Figure 6:**
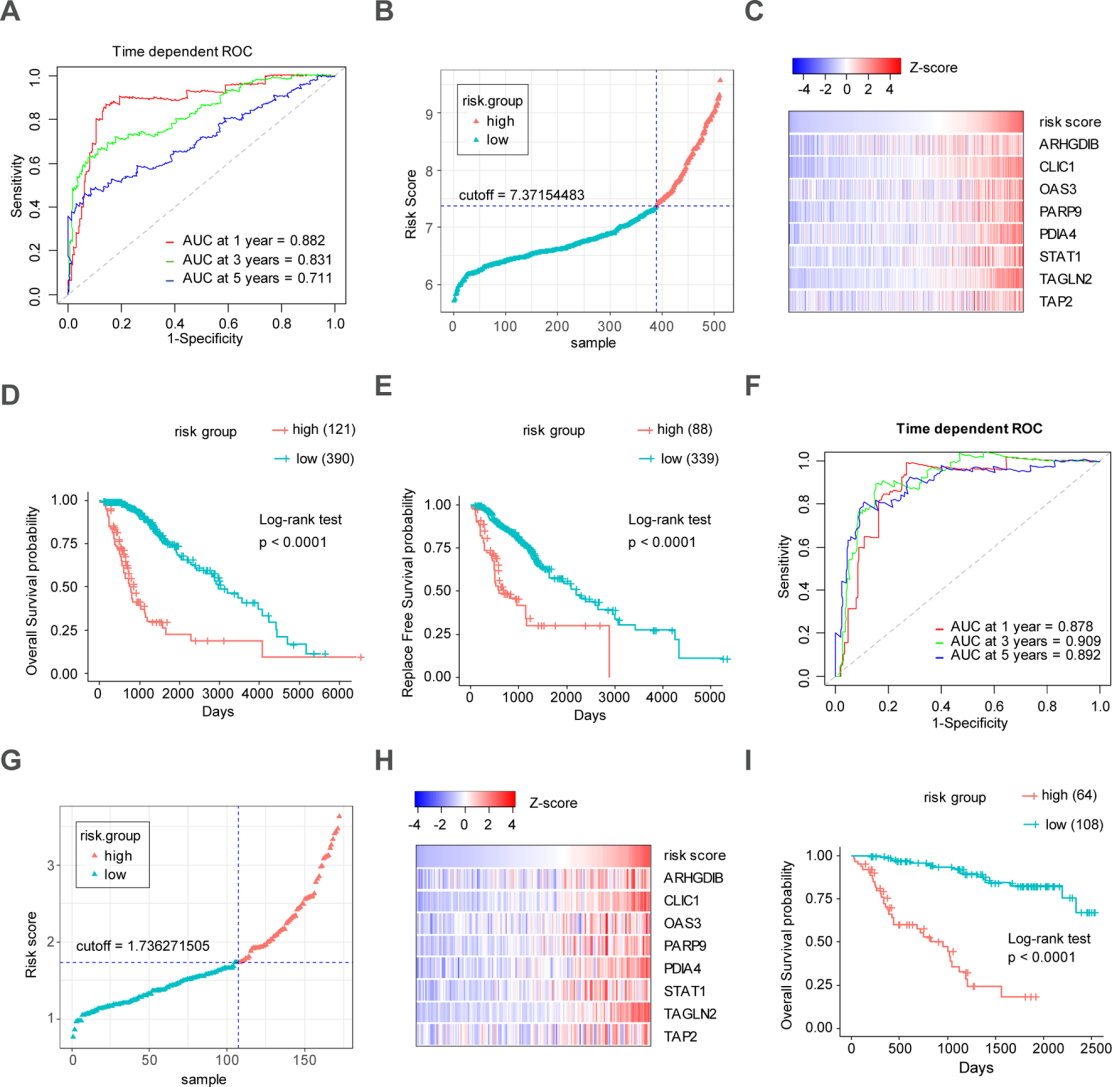
Construction and validation of the eight-gene prognostic model in LrGG cohorts. **(A)** Time-dependent ROC curves indicated good performance of the eight-gene prognostic model in the TCGA cohort. **(B)** The distribution of risk scores in TCGA cohort. **(C)** Heatmap of the model genes in TCGA dataset; the expression of eight genes was transformed by the z-score. **(D)** Kaplan-Meier curve for OS in TCGA LrGG cohort stratified by the eight-gene model as high- and low-risk. **(E)** Kaplan-Meier curve for RFS in TCGA LrGG cohort stratified by the eight-gene model into high- and low-risk. **(F)** Time-dependent ROC curves indicated good performance of our prognostic model in the CGGA cohort. **(G)** The distribution of risk scores in the CGGA cohort. **(H)** Heatmap of the model genes in the CGGA dataset; the expression of eight genes was transformed by the z-score. **(I)** Kaplan-Meier curve for OS in the CGGA LrGG cohort stratified by the eight-gene model into high- and low-risk.

Through the LASSO regression method, eight genes, including *ARHGDIB*, *CLIC1*, *OAS3*, *PARP9*, *PDIA4*, *STAT1*, *TAGLN2*, and *TAP2*, were selected to construct the prognostic risk score model (**Figures 5A, B**). The formula for calculating risk score was as follows: risk score = (-0.1880 * expression level of A*RHGDIB*) + (0.3131 * expression level of *CLIC1*) + (0.0546 * expression level of *OAS3*) + (0.2527 * expression level of *PARP9*) + (0.1052 * expression level of *STAT1*) + (0.1776 * expression level of *PDIA4*) + (0.2479 * expression level of *TAGLN2*) + (-0.2525 * expression level of *TAP2*). To evaluate the predictive accuracy and sensitivity of our prognostic model, a time-dependent ROC curve analysis was performed. In the TCGA cohort, the area under the ROC curve (AUC) of this model for 1-, 3-, and 5-year OS was 0.882, 0.831, and 0.711, respectively (**Figure 6A**). Next, based on the optimal cutoff value (7.37154483) for the 5-year OS, our model presented good sensitivities and specificities to predict the 1-year (sensitivity = 0.906, specificity = 0.806), 3-year (sensitivity = 0.668, specificity = 0.889), and 5-year (sensitivity = 0.486, specificity = 0.915) OS, and we divided the patients in the TCGA LGG cohort into the high-risk group and low-risk group (**Figure 6B**). The heatmap showed that these eight genes were remarkably overexpressed in the high-risk group (**Figure 6C**). Additionally, differential expression analysis *via* the GEPIA revealed that all eight genes were significantly upregulated in LrGG samples compared with those in normal brain tissues (**Supplementary Figure S3**). To evaluate the prediction ability of risk score on OS and Relapse Free Survival (RFS) of LrGG patients, the Kaplan-Meier analysis was performed. The results showed that patients in the high-risk group had significantly shorter OS and RFS than those in the low-risk group based on the TCGA dataset (**Figures 6D–E**). Furthermore, these results were well validated in the CGGA LrGG cohort. In the CGGA cohort, the AUC of this model for 1-, 3-, and 5-year OS was 0.878, 0.909, and 0.892, respectively (**Figure 6F**). Using the same formula and standard for grouping, our model also presented good sensitivities and specificities for predicting the 1-year (sensitivity = 0.991, specificity = 0.706), 3-year (sensitivity = 0.907, specificity = 0.820), and 5-year (sensitivity = 0.810, specificity = 0.891) OS, and the patients were divided into high-risk and low-risk group; the patients in the low-risk group showed a significantly better outcome than those in the high-risk group (**Figures 6G–I**) in the CGGA cohort. Taken together, these results suggest the potential favorable efficiency as well as general applicability of this eight-gene model in predicting short- and long-term prognosis in patients with LrGG.

### The Prognostic Model Is an Independent Predictor and a Valuable Hierarchical Factor

**Figure 7:**
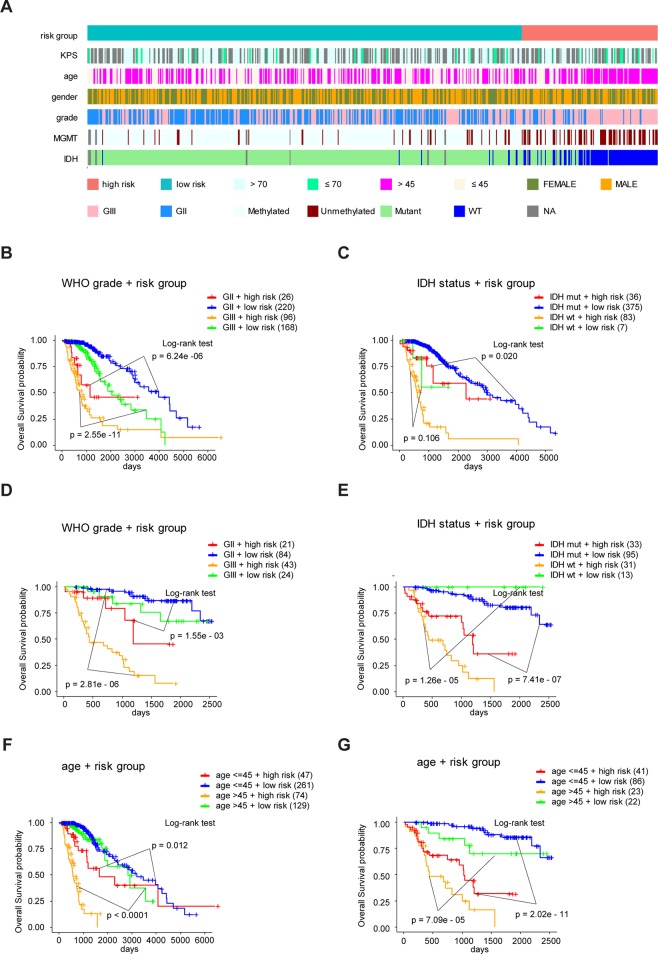
Eight-gene signature performance in clinicopathological and molecular subgroups in TCGA and CGGA cohorts. **(A)** Comparison of the eight-gene signature with the clinicopathological and molecular features of LrGG in TCGA cohort. **(B)** Kaplan-Meier survival curves for OS between grade II and grade III patients with high-risk and low-risk in TCGA cohort. **(C)** Kaplan-Meier survival curves for OS between IDH-Wt and IDH-Mut patients with high-risk and low-risk in TCGA cohort. **(D)** Kaplan-Meier survival curve for OS between grade II and grade III patients with high-risk and low-risk in the CGGA cohort. **(E)** Kaplan-Meier survival curve for OS between IDH-Wt and IDH-Mut patients with high-risk and low-risk in the CGGA cohort. **(F)** Kaplan-Meier survival curves for OS between age subgroups with high-risk and low-risk in TCGA cohort. **(G)** Kaplan-Meier survival curves for OS between age subgroups with high-risk and low-risk in CGGA cohort.

To study the association between the risk groups classified based on our risk score and the clinicopathological factors (age, gender, histological grade) and molecular characteristics (IDH status and MGMT promoter methylation status) in LrGG, we evaluated the statistical difference of the latter parameters between high and low-risk groups using the chi-square test. The result demonstrated that these parameters, apart from gender, were significantly different in patients from high- and low-risk groups in both the TCGA and CGGA datasets (**Figure 7A**, **Table 3**). To identify if our prognostic model was an independent prognostic factor in LrGG, both univariate and multivariate Cox analysis were performed. After adjusting with the abovementioned clinicopathological and molecular characteristics, our prognostic model was still a significantly predictive factor (**Table 4** and **Table 5**). Furthermore, stratified survival analysis was performed to evaluate the prognostic values of this prognostic model in different subgroups of LrGG. In the TCGA cohort, the results showed that this model stratified patients with grade II or grade III glioma well in the OS. When both grade and risk scores were considered, grade II glioma patients within the low-risk group showed the best outcomes, whereas grade III glioma patients belonging to the high-risk group presented the worst prognosis (**Figure 7B**). Similarly, our model also stratified patients in different IDH subgroups, especially the IDH-Mut subgroup, remarkably well in the OS. When both IDH status and risk scores were considered, patients harboring IDH-Wt in the high-risk group had the worst outcome, whereas IDH-Mut patients in the low-risk group had the best prognosis (**Figure 7C**). Furthermore, these results were well validated in the CGGA cohort (**Figures 7D, E**). Our model also stratified patients in different age subgroups (age >45 or age ≤45) notably well in both the TCGA and CGGA cohorts. When both age and risk scores were considered, high-risk patients with age > 45 had the worst OS, whereas low-risk patients with age ≤45 had the best OS (**Figures 7F, G**). Collectively, these data suggest that our prognostic model is an independent prognostic factor as well as a valuable factor for stratification in LrGG.

**Table 3:** Clinical characteristics of LrGG patients within different risk groups in TCGA and CGGA cohorts.

	TCGA Risk group			CGGA Risk group		
	High (121)	Low (390)	p.value (χ^2^ test)	High (64)	Low (108)	p.value (χ^2^ test)
MGMT promoter methylation						
Methylated	63	353	5.27E-23	–	–	–
Unmethylatd	56	29		–	–	
IDH status						
Mutant	36	375	1.02E-62	33	95	3.26E-07
WT	83	7		31	13	
Gender						
Female	55	173	0.915	24	43	0.889
Male	66	217		40	65	
Age						
>45	74	129	6.37E-08	23	22	0.039
≤45	47	261		41	86	
WHO grade						
GIII	95	169	3.18E-11	43	24	1.32E-08
GII	26	220		21	84	

**Table 4:** Univariate and multivariate Cox analysis in TCGA LrGG cohort.

	Univariate analysis		Multivariate analysis	
	HR (95% CI)	P value (Wald test)	HR (95% CI)	P value (Wald test)
MGMT promoter	2.981	<0.001	1.010	0.970
(Unemethylated vs. Methylated)	(2.033-4.370)		(0.610-1.671)	
IDH status	9.243	<0.001	3.368	0.001
(Wt vs. Mutant)	(6.261-13.650)		(1.607-7.059)	
Gender	1.093	0.625	–	–
(Male vs Female)	(0.766-1.559)		–	–
Age	0.359	<0.001	0.531	0.003
(≤45 vs >45)	(0.248-0.519)		(0.352-0.802)	
WHO grade	0.300	<0.001	0.448	<0.001
(GII vs. GIII)	(0.203-0.443)		(0.288-0.699)	
Risk Score	0.187	<0.001	0.490	0.026
(Low vs. High)	(0.131-0.268)		(0.261-0.920)	

**Table 5:** Univariate and multivariate Cox analysis in CGGA LrGG cohort.

	Univariate analysis		Multivariate analysis	
	HR (95% CI)	P value (Wald test)	HR (95% CI	P value (Wald test)
IDH group	3.735	<0.001	1.059	0.875
(Wt vs. Mutant)	(2.115-6.595)		(0.521-2.152)	
Gender	1.077	0.799	–	–
(Male vs. Female)	(0.608-1.908)			
Age	0.33	<0.001	0.547	0.066
(≤45 vs. >45)	(0.188-0.581)		(0.288-1.041)	
WHO grade	0.166	<0.001	0.350	0.003
(GII vs. GIII)	(0.090-0.306)		(0.175-0.701)	
Risk Score	0.094	<0.001	0.1672	<0.001
(Low vs. High)	(0.048-0.182)		(0.081-0.345)	

### The Prognostic Model Predicts the Sensitivity for Radio- and Chemotherapy

**Figure 8:**
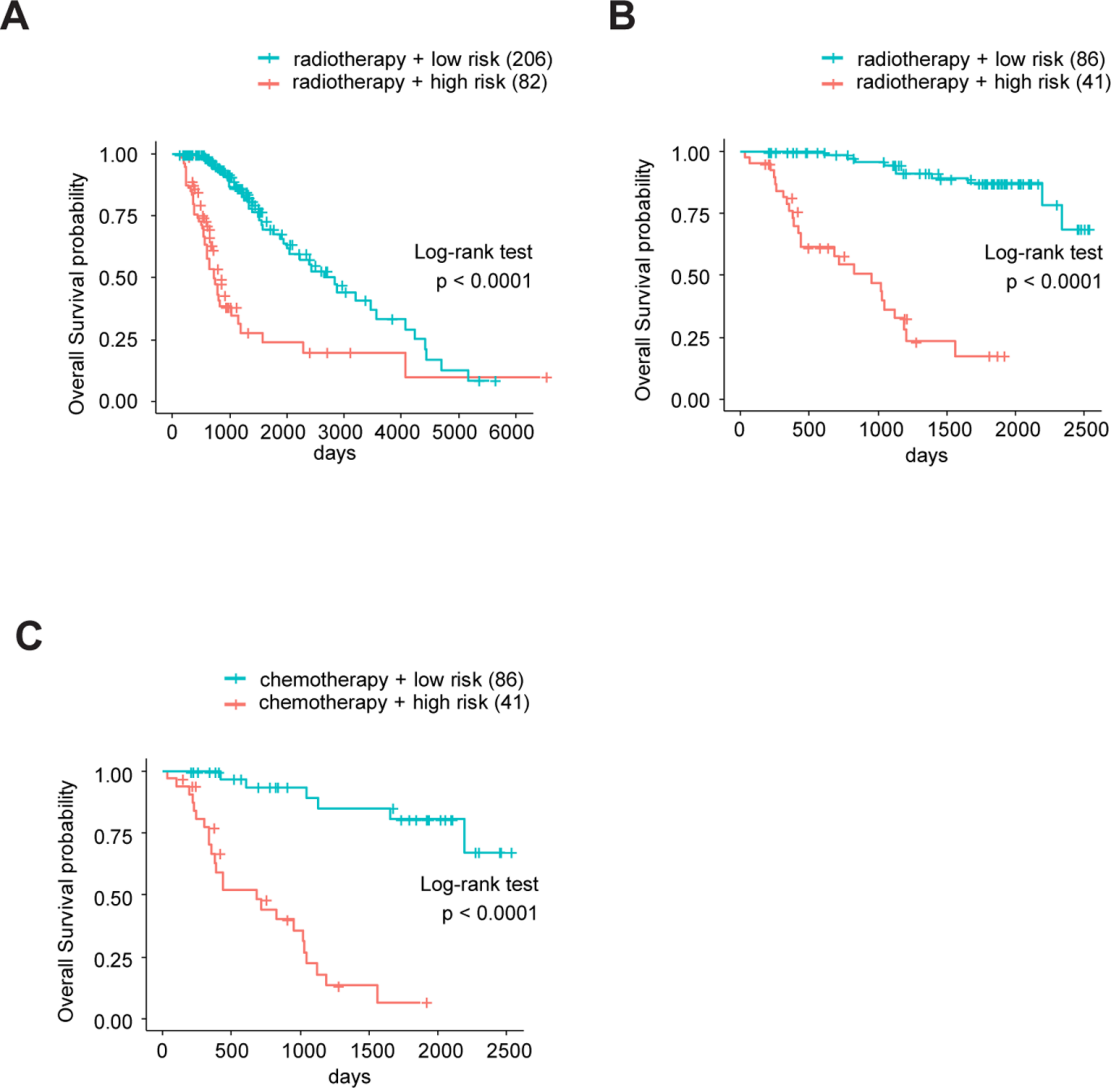
The eight-gene model and treatments (radio- and chemotherapy). **(A)** Kaplan-Meier survival curves for OS between patients who underwent radiotherapy in high-risk and low-risk groups in TCGA cohort. **(B)** Kaplan-Meier survival curves for OS between patients who underwent radiotherapy in high-risk and low-risk groups in CGGA cohort. **(C)** Kaplan-Meier survival curves for OS between patients who underwent chemotherapy in high-risk and low-risk groups in CGGA cohort.

Sine TME plays a substantial role in treatment responses and, as our signature was based on eight TME-related genes, we wondered whether this model could predict the sensitivity of LrGG for radio- and chemotherapy. Through Kaplan–Meier curve analysis, we found that LrGG patients who underwent radiotherapy belonging to the low-risk group had longer OS than those belonging to the high-risk group in the TCGA cohort (**Figure 8A**). Consistently, we observed similar result in the CGGA cohort (**Figure 8B**). For chemotherapy, we found our risk score significantly associated with the methylation status of the MGMT promoter, which can effectively predict the responsiveness of glioma to alkylating agents, the commonly used chemotherapeutic drugs for glioma (Esteller et al., 2000; Hegi et al., 2005; Song et al., 2015). The majority of patients (65.9%) with MGMT promoter unmethylation belonged to the high-risk group and the majority of patients (84.9%) with MGMT promoter methylation belonged to the low-risk group (**Table 3**). Furthermore, Kaplan–Meier curve analysis showed that the prognosis of patients who underwent chemotherapy in the low-risk group was better than that of patient underwent chemotherapy in the high-risk group in the CGGA LrGG cohort (**Figure 8C**). Taken together, these results indicate that our prognostic model have the potential value for predicting the sensitivity of LrGG patient to radio- and chemotherapy.

### The Prognostic Model Correlates With Immune Infiltration in LrGG

**Figure 9:**
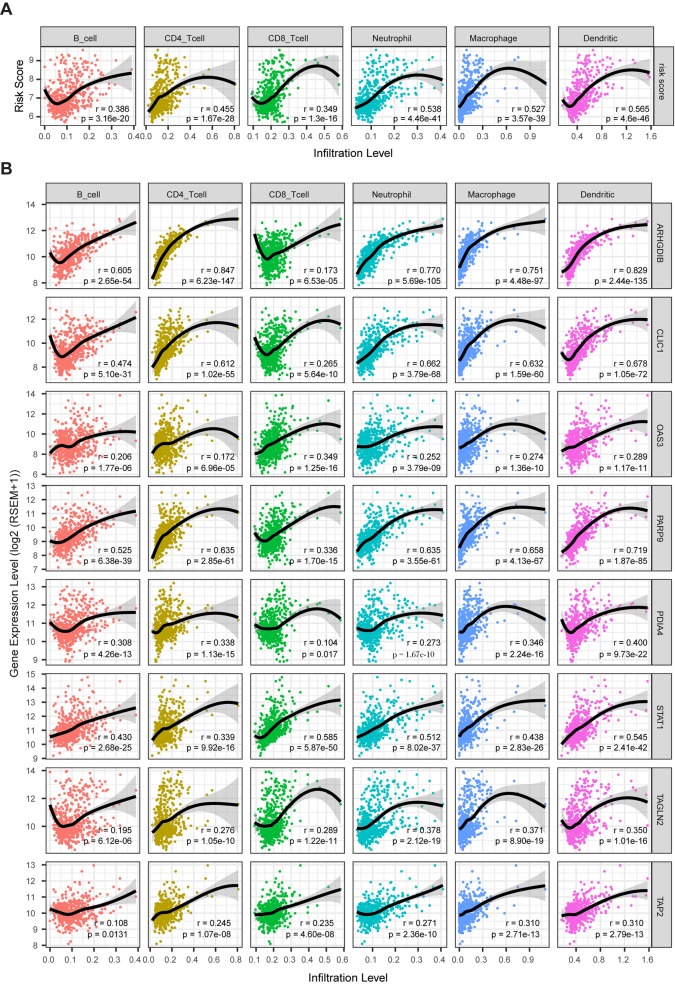
The correlation between model genes as well as risk score and infiltration level of immune cells (Spearman’s r and p, smoother is LOESS, and confidence band is SE). **(A)** The risk score significantly associated with infiltrating levels of immune cells. **(B)** *ARHGD1B*, *CLIC1*, *OAS3*, *PARP9*, *PDIA4*, *STAT1*, *TAGLN2*, and *TAP2* were significantly correlated with infiltrating levels of immune cells.

Clinical research on immunotherapy has confirmed that the tumor-infiltrating lymphocytes within the tumor microenvironment have a predictive value for prognosis and treatment with immunotherapy in cancers (Spencer et al., 2016). Considering that our risk score was based on eight TME-related genes, we investigated whether our risk score was correlated with the infiltrating levels of six immune cells in the TCGA LrGG cohort, which were obtained from TIMER. The results showed that the risk score was significantly correlated with the infiltrating levels of B cells (r = 0.386, p = 3.16e - 20), CD4+ T cells (r = 0.455, p = 1.67e - 28), CD8+ T cells (r = 0.349, p = 1.30e - 16), neutrophils (r = 0.538, p = 4.46e - 41), macrophages (r = 0.527, p = 3.57e - 39), and dendritic cells (r = 0.565, p = 4.60e - 46) in LGG (**Figure 9A**). In addition, the immune and stromal scores of patients within the high-risk group were significantly higher than those of patients within the low-risk group (**Supplementary Figure S4A**). For immune cell infiltration, the infiltrating levels of six immune cells in high-risk patients were remarkably higher than those in low-risk patients (**Supplementary Figure S4B**). Next, we analyzed the correlation between the expression levels of eight TME-related genes and the infiltrating levels of six immune cells. The results showed that the expression of these eight genes showed significantly positive associations with immune cell infiltration (p < 0.05, **Figure 9B**). *ARHGDIB* expression presented a strong correlation with the infiltration levels of CD4+ T cells, neutrophils, macrophages, and dendritic cells (0.751 ≤ r ≤ 0.847), moderate correlation with the infiltrating levels of B cells (r = 0.605), and weak correlation with CD8+ T cell infiltration level (r = 0.173). *CLIC1* expression exhibited weak correlation with CD8+ T cell (r = 0.265) infiltration levels and a moderate correlation with the infiltrating levels of other immune cells (0.474 ≤ r ≤ 0.678). The expression of *OAS3* presented a moderate correlation with the infiltrating levels of CD8+ T cells (r = 0.349) and a weak correlation with the infiltrating levels of other cells (0.172 ≤ r ≤ 0.289). *PARP9* expression was strongly correlated with the infiltrating level of dendritic cells (r = 0.719) and was moderately correlated with the infiltrating level of other immune cells (0.336 ≤ r ≤ 0.658). *STAT1* moderately correlated with the infiltrating levels of all six immune cells (0.339 ≤ r ≤ 0.585). The expression of *PDIA4, TAGLN2*, and *TAP2* presented weak to moderate correlation with the infiltrating levels of all six immune cells (0.104 ≤ r ≤ 0.400). Taken together, these results indicate that our model system is significantly associated with the infiltration level of immune cells in the TME of LrGG.

## Discussion

Glioma is a fatal tumor of the central nervous system, and none of the current available treatments are curative. In recent years, an increasing amount of studies have demonstrated that the tumor microenvironment plays a vital role in in tumor malignancy and responses to treatments including immunotherapy. Thus, a comprehensive understanding of the tumor microenvironment in LrGG might help us find novel predictive biomarkers to guide individual treatment and might provide therapeutic targets to develop effective treatment strategies.

In this study, we used the weighted gene co-expression network analysis (WGCNA) to construct a weighted gene co-expression network in LrGG samples from the TCGA database. Due to several advantages such as reflecting the continuous nature of the underlying co-expression information, providing systems-level insights, and high sensitivity to low abundance and small fold-changes in genes without any information loss, WGCNA has been widely applied in various cancers (Pei et al., 2017; Maertens et al., 2018;Wan et al., 2018). Through principal components analysis, we initially screened out the co-expression modules that significantly correlated with the immune score and stromal score as calculated by the ESTIMATE algorithm. After performing GO enrichment analysis of these modules, we obtained three co-expression modules with a total of 601 genes involved in TME. In gene co-expression networks, hub genes are very important nodes with a maximal information exchange with other genes, and they are located centrally in the module (Langfelder and Horvath, 2008; Yang et al., 2014). Thus, intramodular hub genes were extracted with high gene significance and high intramodular connectivity from the three modules. Finally, we obtained 77 hub genes, of which 74 genes were associated with the OS of LrGG in TCGA database. Importantly, 70 genes were well validated in another independent LrGG cohort from the CGGA database, which further confirmed the reliability of these results. Indeed, some of these 70 genes were reported to be involved in glioma tumorigenesis or as being significant in predicting OS. For example, *TAGLN2* (Transgelin-2), a member of the calponin family of actin-bundling proteins, has been reported to promote the development of glioma, and high *TAGLN2* expression is associated with poor prognosis (Han et al., 2017). *CLIC1* is the first member of the Chloride Intracellular Channel family; its expression has been correlated with poor prognosis and it is known to modulate the cell cycle progression of glioblastoma stem cells (Wang et al., 2012; Gritti et al., 2014). Thus, these results might provide novel insights into the TME of LrGG at the molecular level, and these prognostic genes might act as biomarkers and/or therapeutic targets for the diagnosis, outcome prediction, and treatment of LrGG in the future.

The individual prognosis of LrGG patients varies greatly; however, both the histopathological features and recently established genetic biomarkers such as *IDH*, 1p/19q codeletion, *ATRX*, and *TERT*, of diffuse gliomas often fail to precisely predict a prognosis and completely explain this difference (Boots-Sprenger et al., 2013). Although IDH mutation strongly predicts good prognosis in glioma, most LrGG harbor IDH mutation and its predictive value in LrGG was not as good as that in GBM. On the other hand, adults with IDH-Wt LrGG do not have uniformly poor prognoses, nor is there a uniformly good outcome of LrGG with IDH mutation (Aibaidula et al., 2017; Liu et al., 2019). Thus, the LrGG need to be further stratified. More recently, some genetic prognostic models have been constructed and their predictive performances have been validated in glioma. For example, Zeng et al. (2018) identified and validated a three-gene prognostic signature for LrGG by integrative Analysis of DNA Methylation and Gene Expression data. Yin et al. (2019) selected five genes from differentially expressed genes in glioblastoma to construct a prognostic model with potential in the prognosis prediction of GBM. Kang et al. (2019) developed a 5-CpG signature of miRNA methylation with prognostic values in non-G-CIMP GBM patients. It is thus obvious that the genes within the majority of constructed prognostic models are selected by differential expression analysis. However, a prognostic model based on TME-related genes based on gene co-expression analysis was rarely reported for LrGG. Through differential expression analysis of known immune-related genes between IDH-WT and IDH-Mut LrGG, Qian et al. (2018) revealed that IDH was associated with the regulation of immune microenvironment in LrGG and reported an IDH-associated immune signature. However, focusing on IDH-associated immune-related genes might limit our understanding of TME in LrGG and the clinical value of their signature. In order to comprehensively understand the TME in LrGG, our analysis did not emphasize any known clinicopathological or molecular factors. In the present study, by combining the WGCNA and LASSO methods, we successfully developed a prognostic model with eight TME-related genes for LrGG. Our prognostic model showed a favorable efficiency in predicting both short- and long-term prognoses for LrGG patients. Furthermore, stratified survival analysis demonstrated that this prognostic model still had a prognostic value for patients in different clinicopathological and molecular subgroups. Moreover, our risk model was an independent predictive factor for LrGG after adjusting for clinicopathological and molecular factors. In summary, our risk model has potential value for the prediction of prognoses in LrGG.

Despite many studies indicating that complete neurosurgical resection can effectively prolong the OS rate of LrGG, this is unachievable for most patients due to the invasive characteristics of LrGG, the location of the tumor, and other reasons. Patients with residual tumors might undergo adjuvant radiotherapy and chemotherapy to delay or control tumor progression. However, LrGG exhibits a differential response to radio- and chemotherapy, and some patients cannot even benefit from these treatments (Forst et al., 2014). Thus, identifying LrGG patients who are sensitive to these treatments is especially important. Through Kaplan–Meier curve analysis, we found that our model had the potential value of predicting LrGG patient sensitivity to both radiotherapy and chemotherapy. This might be related to the STAT1 signaling. Notably, we found that our prognostic model consists of three known or presumable targets (*STAT1*, *OAS3* and *TAP2*) and one modulator (*PARP9*) of STAT1 signaling (Samuel, 2001; Zhang et al., 2015; Iwata et al., 2016; Narkwa et al., 2017). Additionally, these four genes were co-expressed in both TCGA and CGGA LrGG cohorts (**Figure 3A** middle, **Supplementary Figures S5A and B**). Furthermore, an increasing amount of studies have indicated that STAT1 is associated with radio- and chemoresistance in multiple tumor entities (Weichselbaum et al., 2008; Khodarev et al., 2012). Indeed, *STAT1* had been reported to be aberrantly expressed in glioblastoma and an overexpression of *STAT1* predicted poor prognosis (Thota et al., 2014). Duarte et al. developed a signature of IFN/STAT1 signaling genes, which presented strong predictive value in the proneural subtype glioblastoma (Duarte et al., 2012). Similarly, our analysis also demonstrated that *STAT1* was aberrantly expressed in LrGG and a high expression of *STAT1* predicted poor prognosis of LrGG patients (**Supplementary Figures S5C, D**). The time-dependent ROC analysis revealed that *STAT1* alone presented favorable efficiency for predicting prognosis of LrGG in both TCGA and CGGA LrGG cohorts (**Supplementary Figures S5E, F**). However, comparing the AUC of our model and *STAT1*, we found our model had better predictive abilities than *STATA1* alone.

In addition, the risk score and expression levels of all eight genes in our model exhibited a significantly positive association with the infiltrating levels of immune cells including B cells, CD4+ T cells, CD8+ T cells, dendritic cells, macrophages, and neutrophils, whereas a relatively low correlation was observed between the gene expression and CD8+ T cells. Accumulating research suggests that the efficiency of immunotherapy relies on an immunogenic TME, and tumor-infiltrating lymphocytes within the TME have a predictive value for treatment with immunotherapy in cancers (Spencer et al., 2016; Gasser et al., 2017). Association with infiltrating immune cells could be a useful criterion for selecting putative cancer vaccine targets (Li et al., 2016). Additionally, it is confirmed that some of these genes are involved in the regulation of immune cells. The cleaved form of *ARHGDIB* (also called RhoGDIβ), cleaved by caspase-3 at Asp19, plays a role in the PMA−induced differentiation of THP−1 cells to macrophages (Ota et al., 2017). *CLIC1*, intracellular chloride channel protein 1, regulates macrophage phagosomal functions and regulates the dendritic cell processing of antigens for presentation to antigen-specific T cells (Salao et al., 2016). Furthermore, all of these eight genes were aberrantly expressed in LrGG samples compared to normal brain tissues. Taken together, our model system might have a predictive value for immunotherapy, and these 8 genes might be potential immunotherapy targets that deserve further study.

There are several limitations to our study. First, because of the limited clinical information of patients in public databases, we were unable to perform stratified survival analysis in more subgroups. Second, the performance of our prognostic model should be validated in more LrGG datasets. Third, all the results were based on public datasets and should be further confirmed by actual experiments.

**Figure 10:**
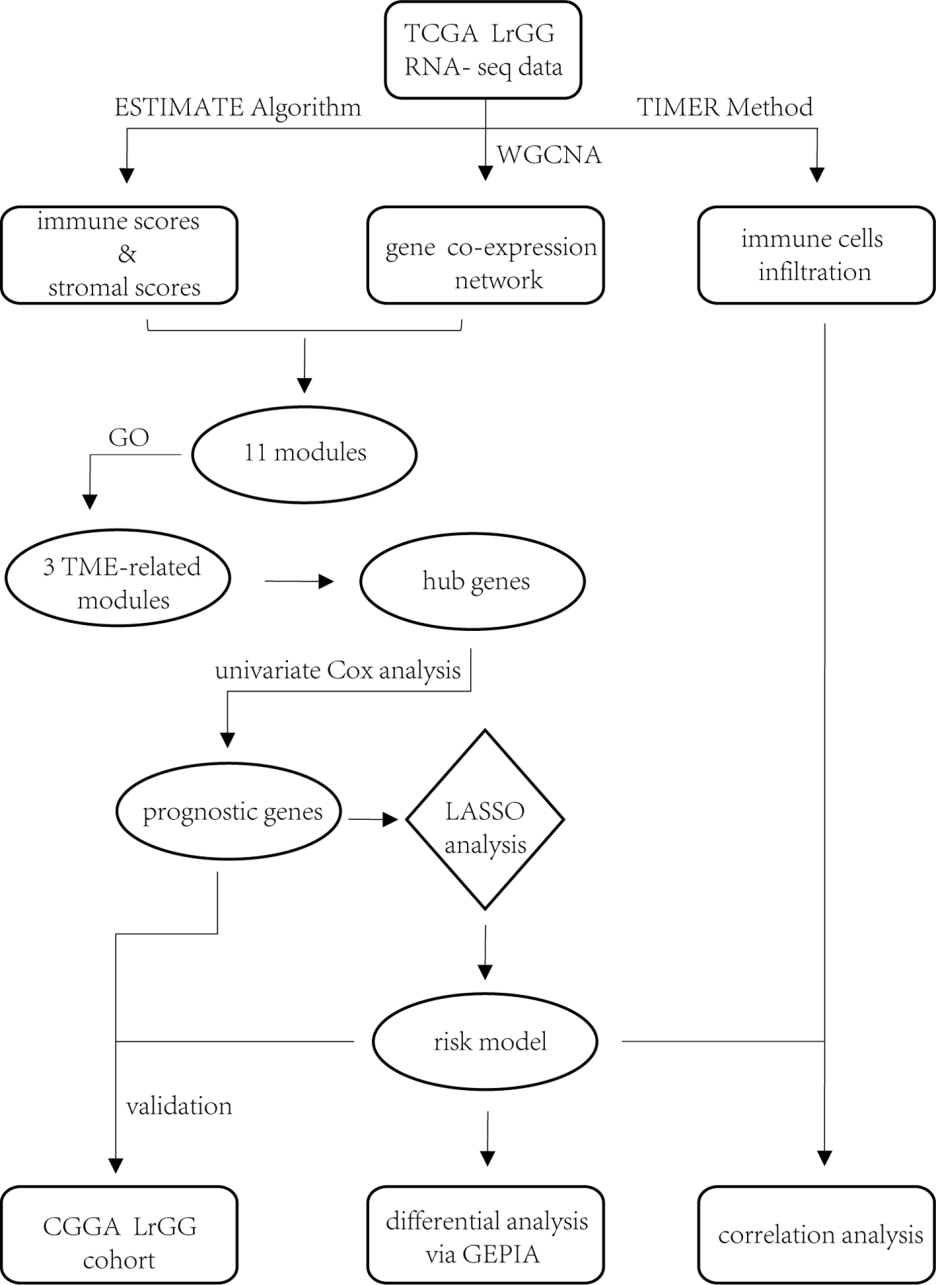
Workflow of the current work.

In summary, we identified 74 prognostic hub genes from three TME-related gene co-expression modules and selected eight of these to construct a prognostic model (**Figure 10**). Time-dependent ROC curves analysis and survival analysis demonstrated that our model exhibited excellent efficiency for predicting the prognosis of LrGG and was a valuable hierarchical marker. Furthermore, our model had the potential value of predicting sensitivity for radio- and chemotherapy. In addition, these eight genes within our model were aberrantly expressed and were significantly associated with the infiltrating level of immune cells in LrGG, indicating their potential as targets for immunotherapy.

## Data Availability Statement

Publicly available datasets were analyzed in this study. This data can be found here: https://xenabrowser.net/datapages/?cohort=TCGA%20Lower%20Grade%20Glioma%20 ;https://gdac.broadinstitute.org/; http://www.cgga.org.cn/download.jsp.

## Author Contributions

JS analyzed the data, performed computational coding, and wrote the manuscript. QM and WL were involved in the design of the study. KX, YL, QX, GP, and JY collected the data. QL was involved in the design of the study, the manuscript review and editing, and the supervision of the entire work. All authors have approved the final manuscript.

## Funding

This study was supported by grants from the National Natural Science Foundation of China (No.81802974)

## Conflict of Interest

The authors declare that the research was conducted in the absence of any commercial or financial relationships that could be construed as a potential conflict of interest.
