# Supplementary material for: Identification of a Tumor Microenvironment-Related Eight-Gene Signature for Predicting Prognosis in Lower-Grade Gliomas
Source: Front Genet. 2019 Nov 15;10:1143. doi: 10.3389/fgene.2019.01143 (PMC6872675; doi:10.3389/fgene.2019.01143)
Supplement: Supplementary file 1 [file DataSheet_1.zip › supplementary table captions and figure legends.docx]

Supplementary Material

# Supplementary Table Captions

**Supplementary Table S1.** GO analysis of 3 TME-related co-expression modules (green, magenta, and salmon).

**Supplementary Table S2.** GO analysis of other 8 co-expression modules.

**Supplementary Table S3.** GO and KEGG pathway enrichment analysis of 70 prognostic hub genes.

# Supplementary Figure legends

**Supplementary Figure S1.** Clustering dendrogram of the TCGA LrGG samples based on their Euclidean distance

**Supplementary Figure S2.** GO analysis of 3 TME-related co-expression modules (P value was adjusted by Benjamini and Hochberg method)

A The top10 terms of biological process of GO enrichment analysis of genes in green module.

B The top10 terms of molecular function terms of GO enrichment analysis of genes in green module.

C The top10 terms of biological process of GO enrichment analysis of genes in magenta module.

D The top10 terms of molecular function terms of GO enrichment analysis of genes in magenta module.

E The top10 terms of biological process of GO enrichment analysis of genes in salmon module.

F The top10 terms of molecular function terms of GO enrichment analysis of genes in salmon module.

**Supplementary Figure S3.** Differential expression analysis of the eight genes within our prognostic model via the GEPIA

A *ARHGDIB* was overexpressed in LrGG compared to normal brain tissues.

B *CLIC1* was overexpressed in LrGG compared to normal brain tissues.

C *OAS3* was overexpressed in LrGG compared to normal brain tissues.

D *PARP9* was overexpressed in LrGG compared to normal brain tissues.

E *PDIA4* was overexpressed in LrGG compared to normal brain tissues.

F *STAT1* was overexpressed in LrGG compared to normal brain tissues.

G *TAGLN2* was overexpressed in LrGG compared to normal brain tissues.

H *TAP2* was overexpressed in LrGG compared to normal brain tissues.

*** P < 0.001, T means tumor tissue (red color), N means normal brain tissue (gray color), one-way ANOVA test.

**Supplementary Figure S4.** Distribution of immune score, stromal score and infiltrating levels of immune cells in LrGG of different risk groups.

A Both immune and stromal scores in high-risk group LrGG were significantly higher than those in low-risk group LrGG.

B The infiltration levels of 6 immune cells in LrGG divided into high-risk group were significantly higher than that in LrGG divided into low-risk group.

*** P < 0.001, t-test

**Supplementary Figure S5.** Correlation among 4 STAT1 signaling genes and *STAT1* associated with prognosis of LrGG in TCGA and CGGA datasets

A-B Visualization of correlation among *STAT1*, *OAS3*, *TAP2* and *PARP9* in TCGA and CGGA LrGG cohorts using corrplot package (Pearson’s r, p value < 0.001, methods included “pie” (upper) and “number” (lower), The “pie” was color-coded by correlation according to the color legend, which decreased in size from red to blue).

C-D Kaplan‐Meier survival curves showed that *STAT1* associates with prognosis of LrGG in TCGA and CGGA datasets.

E-F Time dependent ROC analysis of *STAT1* in the TCGA and CGGA LrGG cohorts.
