# Supplementary figures and images for: Identification of a Tumor Microenvironment-Related Eight-Gene Signature for Predicting Prognosis in Lower-Grade Gliomas
Source: Front Genet. 2019 Nov 15;10:1143. doi: 10.3389/fgene.2019.01143 (PMC6872675; doi:10.3389/fgene.2019.01143)

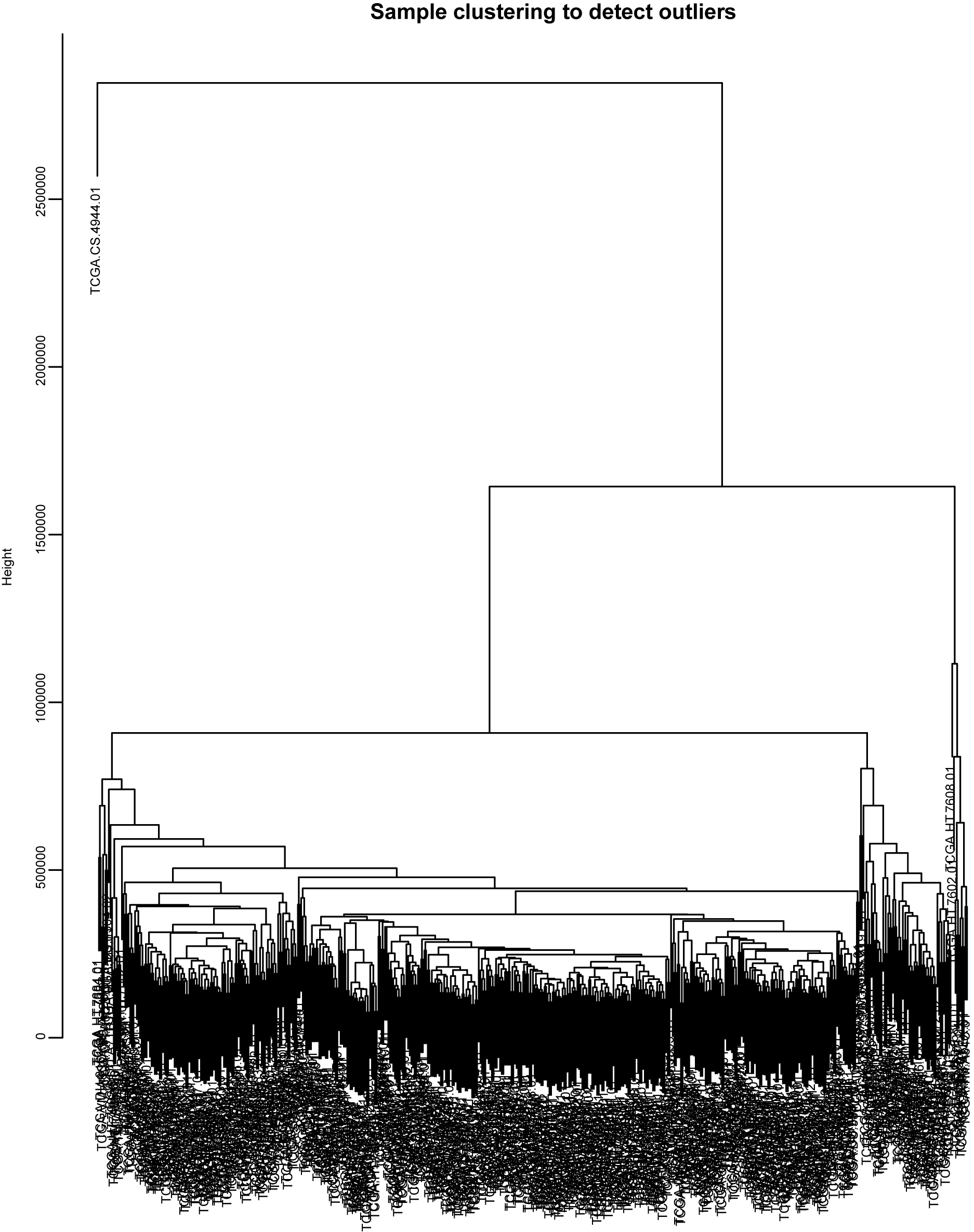

Supplement: Supplementary file 2 [file Image_1.tif]

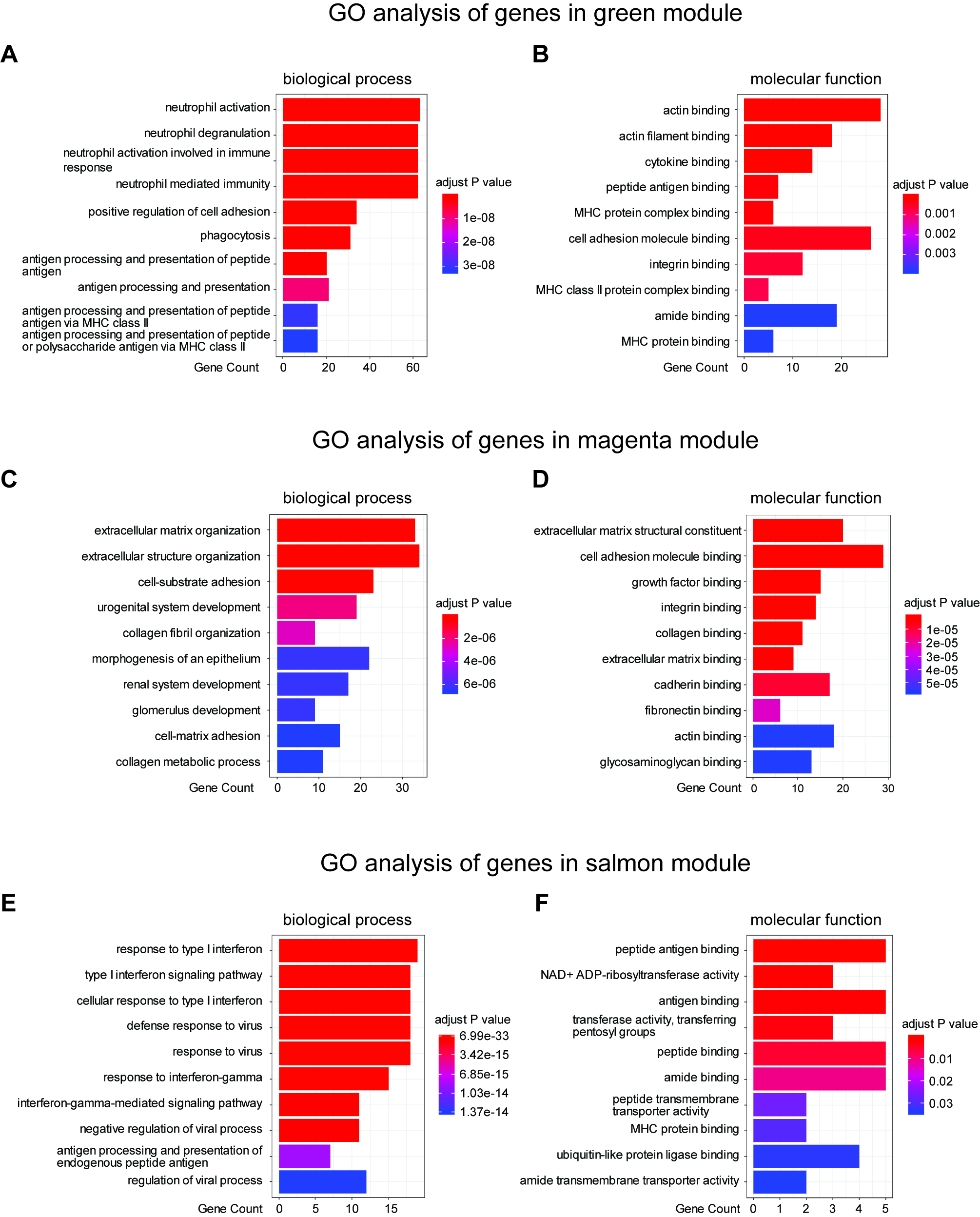

Supplement: Supplementary file 3 [file Image_2.tif]

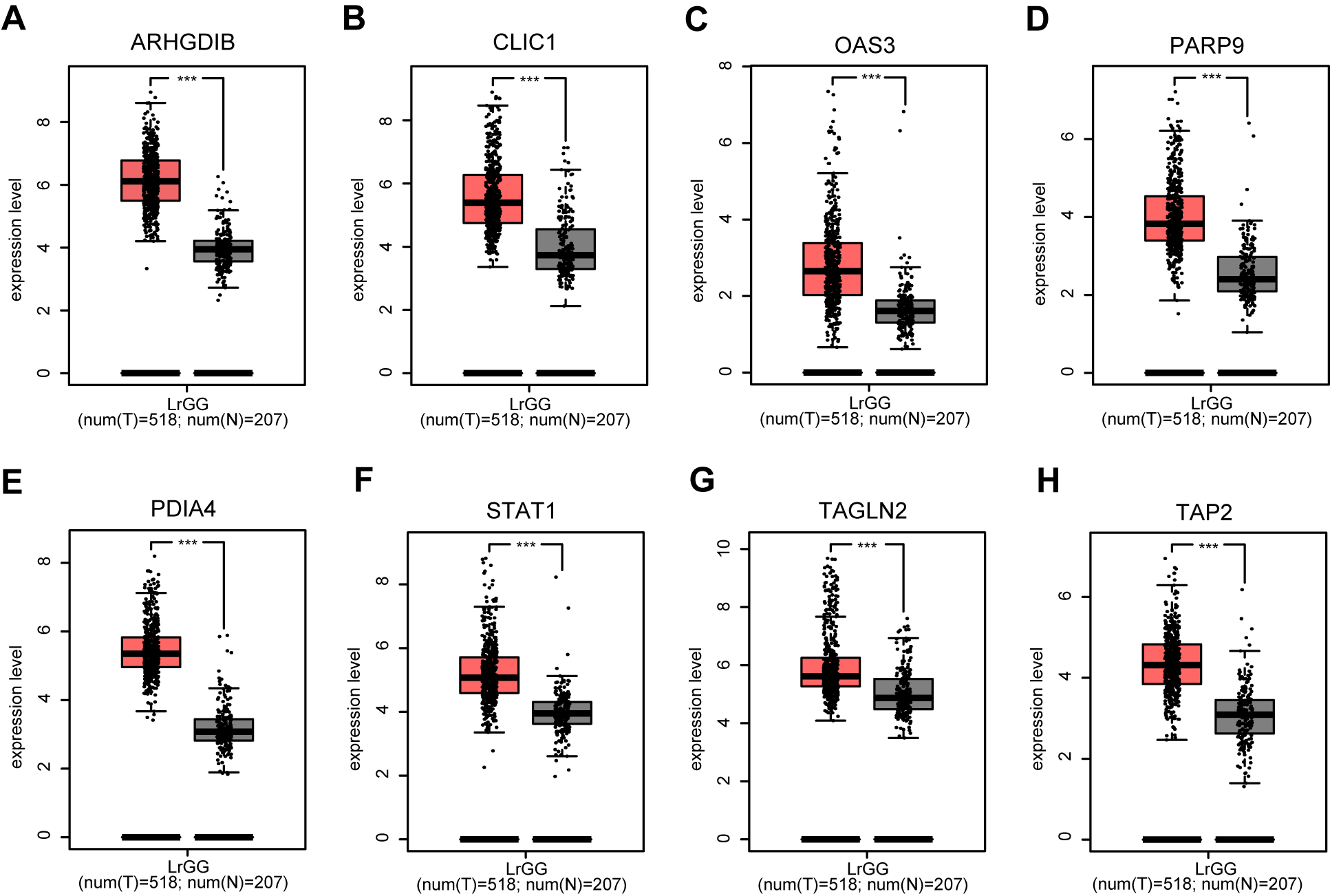

Supplement: Supplementary file 4 [file Image_3.tif]

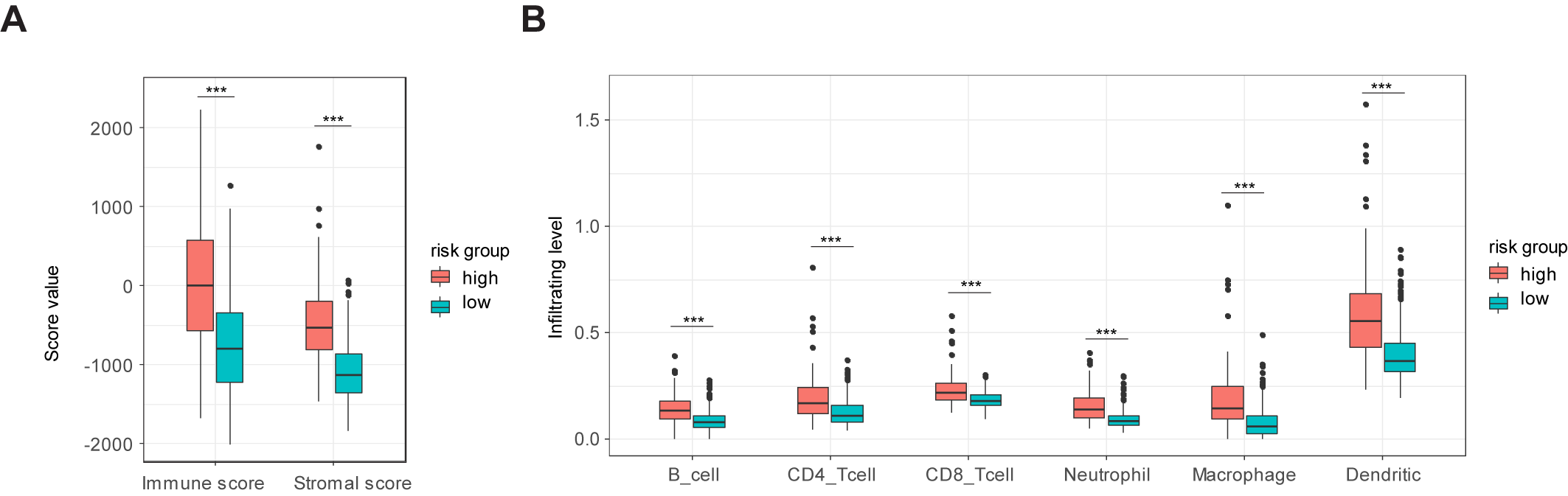

Supplement: Supplementary file 5 [file Image_4.tif]

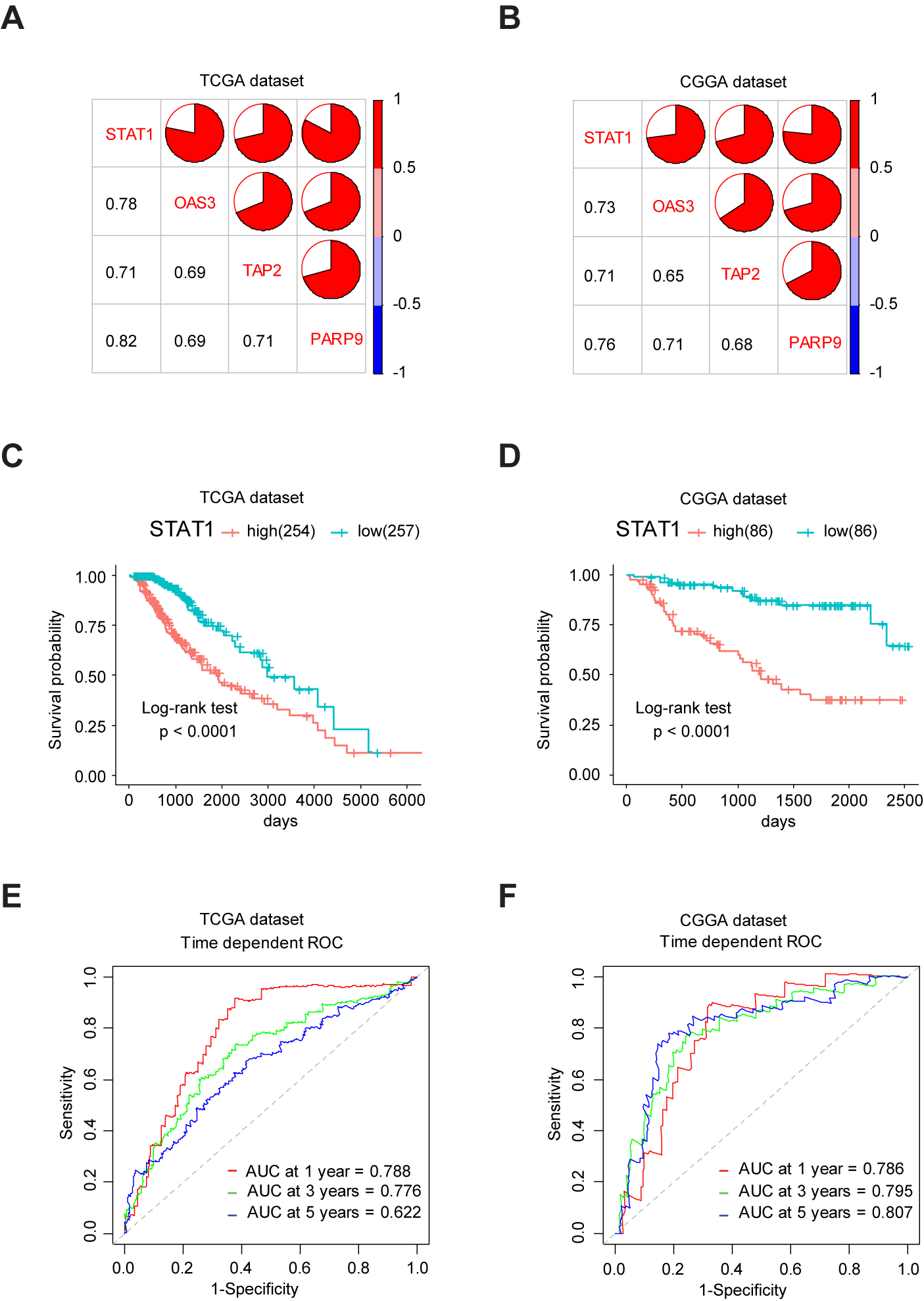

Supplement: Supplementary file 6 [file Image_5.tif]
